# Visual acuity and its postoperative outcome after transsphenoidal adenoma resection

**DOI:** 10.1007/s10143-020-01408-x

**Published:** 2020-10-10

**Authors:** Vicki M. Butenschoen, Nina Schwendinger, Alexander von Werder, Stefanie Bette, Maximilian Wienke, Bernhard Meyer, Jens Gempt

**Affiliations:** 1 Department of Neurosurgery, Klinikum rechts der Isar, Technical University Munich, Ismaningerstr. 22, 81675 Munich, Germany; 2 II. Medizinische Klinik und Poliklinik, Klinikum rechts der Isar, Technische Universität München, Munich, Germany; 3 Abteilung für Diagnostische und Interventionelle Radiologie, Klinikum Augsburg, Augsburg, Germany

**Keywords:** Pituitary adenoma, Transsphenoidal surgery, Visual acuity

## Abstract

Transsphenoidal surgery (TSS) represents the gold standard of pituitary adenoma resection, providing a safe and minimal invasive treatment for patients suffering from symptoms of mass effect. The aim of this study is to analyze the postoperative improvement of visual function after adenoma resection and to identify prognostic factors for the postoperative clinical recovery. We performed a retrospective analysis of all consecutive patients treated via a transsphenoidal approach for pituitary adenomas from April 2006 to December 2019 in a high-volume neurosurgical department. Our primary outcome was postoperative visual acuity and visual field impairment; the clinical findings were followed up to 3 months after surgery and correlated with clinical and radiographic findings. In total, 440 surgeries were performed in our department for tumors of the sella region in a time period of 13 years via transsphenoidal approach, and 191 patients included in the analysis. Mean age was 55 years, and 98% were macroadenomas. Mean preoperative visual acuity in patients with preoperative impairment (*n* = 133) improved significantly from 0.64/0.65 to 0.72/0.75 and 0.76/0.8 (right eye R/left eye L) postoperatively and at 3 months follow-up (*p* < 0.001). Visual acuity significantly depended on Knosp classification but not Hardy grading. The strongest predictor for visual function recovery was age. Transsphenoidal pituitary tumor resection remains a safe and effective treatment in patients with preoperative visual impairment. It significantly improves visual acuity and field defects after surgery, and recovery continues at the 3 months follow-up examination.

## Introduction

Pituitary adenomas present benign lesions with a close spatial relationship to the optic chiasm. Larger tumors growing beyond the sella can affect peripheral vision and even cause elevated intracranial pressure [16, 26, 27]. While the indication for surgical resection of nonfunctioning incidentalomas is often a matter of debate and varies from its timing and treatment strategy [23, 24], adenomas leading to visual impairment are usually treated surgically, in order to improve or halt further progression of vision loss [7, 26] as longer duration of the symptoms has been shown to lead to worse visual outcomes after surgery [26].

Although the transnasal resection of sellar tumors is regarded a safe and efficient treatment option, complications may occur ranging from minor headaches to severe carotid artery hemorrhage and even death [2, 3, 8, 15]. Factors influencing the postoperative outcome as well as the complication rate include age [14, 32, 35], body mass index (BMI) [9], number of surgeries [15], and the surgical approach used [18, 29, 34] as well as tumor size and sinus suprasellar growth [16]. Typical vision changes and field defects include the bitemporal hemianopsia, leading to binocular vision difficulties [28]. The visual postoperative outcome and recovery are known to be favorable, although influencing factors are currently discussed such as preoperative deficits, tumor size und tumor location, age, duration of symptoms, und tumor recurrence [6, 22, 31].

Our study aims to review the postoperative outcome of patients suffering from visual deficits and identify prognostic factors influencing the improvement, stagnation, or even worsening of visual acuity postoperatively and at 3 months follow-up.

## Methods

We conducted a retrospective analysis of all consecutive patients treated for sella turcica pituitary adenoma from April 2006 to December 2018 in our neurosurgical department through a transnasal transsphenoidal approach. Inclusion criteria were adenoma of the sella region, complete data available, age > 18 years, transsphenoidal transnasal operation performed, minimum follow-up time with assessment of visual acuity, and perimetric assessment at 3 months.

Demographic factors such as gender, age, and Karnosfky Performance Status Scale (KPS) as well as comorbidities were retrieved from patient files. We reviewed preoperative imaging (magnetic resonance imaging MRI, computed tomography CT) and obtained detailed information on the preoperative and postoperative visual acuity and visual field defects (mapping of the visual field using a threshold static automated perimetry).

Surgical data included microscopic vs. endoscopic approach, length of operation (minutes), and intraoperative occurrence of cerebrospinal fluid (CSF) leaks. Visual acuity and field defects were examined on postoperative day (POD) 6 as well as clinical status via KPS. Extent of resection (EOR) was classified in gross total resection (GTR) vs. partial resection (PR). Postoperative complications (transient or persistent diabetes insipidus, CSF leaks with the need of surgical revision, neurological deterioration) and tumor histology were assessed. The minimum follow-up time was 3 months.

We performed statistical correlation analyzes using SPSS Version 26.0.0.0 and R Version 3.6.3, adjusting for age and gender by conducting multivariate factor evaluation and controlling for possible confounders. Subgroup analysis was performed for patients suffering from preoperative visual acuity impairment; chi-square and Fisher’s exact test were used for significance testing.

**Fig. 1:**
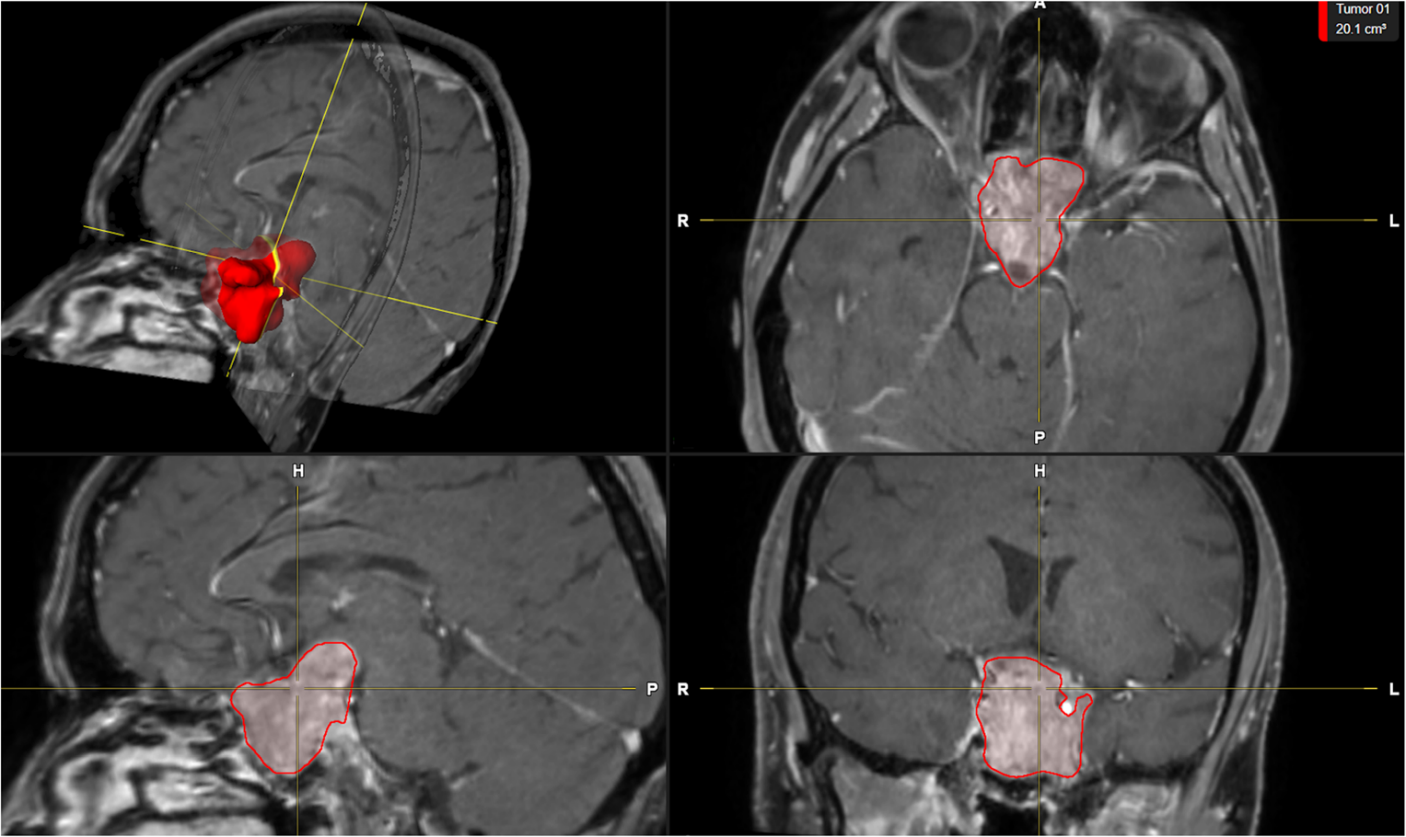
Volumetric assessment of tumor size (red), cavernous sinus invasion (in this case Knosp grade 4), and tumor invasiveness through the sella floor (Hardy classification, in this case classified as class 3) using the Brainlab software on preoperative MRI

Tumor volume and tumor extension were measured and classified by Knosp et al. for cavernous sinus invasion [17] and by the Wilson-Hardy classification to describe tumor invasiveness through the sella floor [12], as well as the pituitary gland delineation (Fig. 1). Preoperative tumor volume was manually segmented using iPlan Net (iPlan Net Cranial 3.0, Brainlab AG, Munich) by a neuroradiologist (S.B., 8 years of experience) and a neurosurgeon (V.B, 5 years of experience). To assess inter-rater reliability, the intraclass correlation coefficient (ICC) was calculated for 50 randomly chosen subjects as described before [11].

## Results

### Patient population

**Fig. 2:**
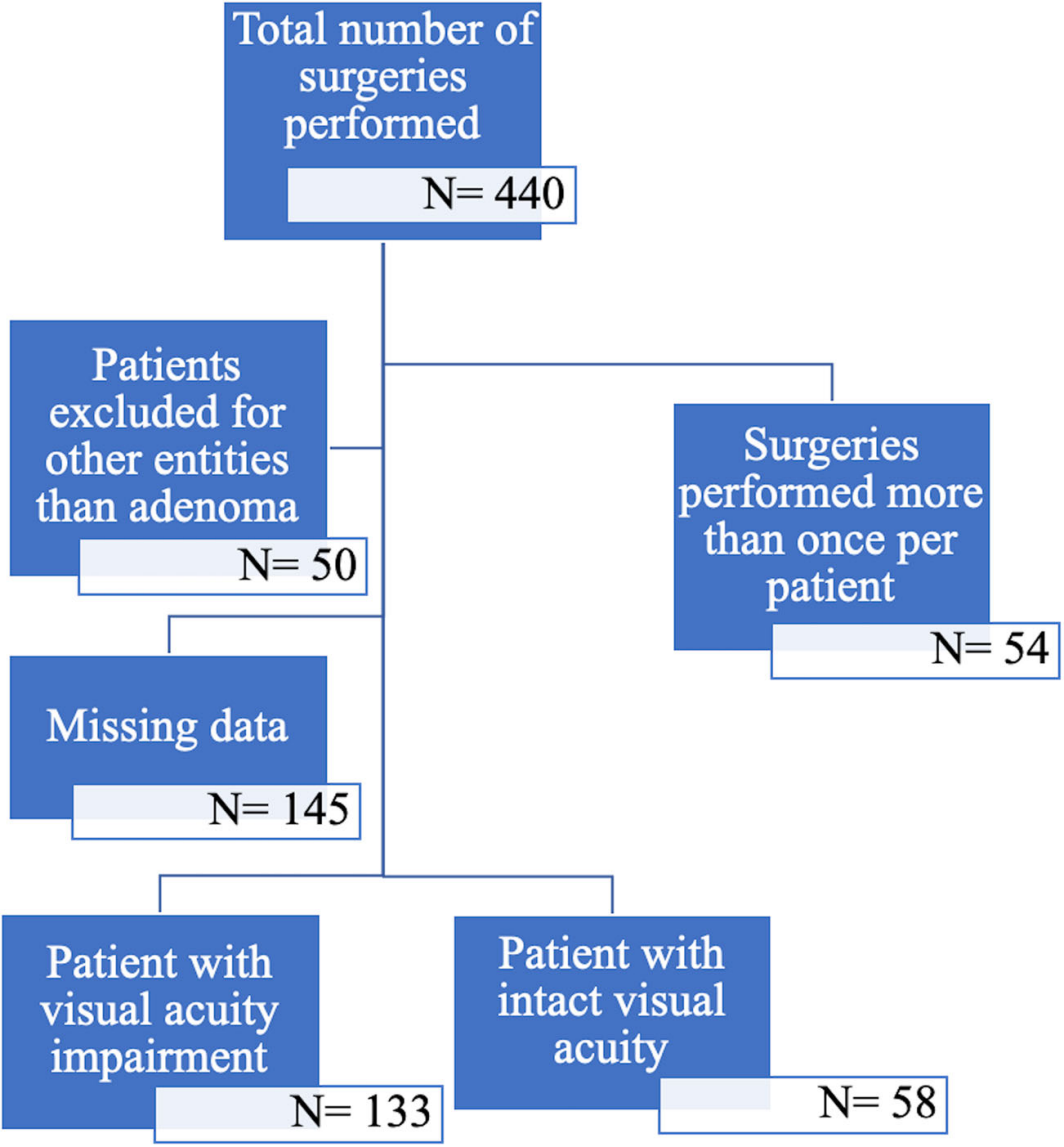
Flowchart describing the number of patients meeting the inclusion and exclusion criteria and describing the clinical symptoms at first consultation grouped by type of visual impairment

In total, 440 transsphenoidal transnasal surgeries were performed in 386 patients treated for tumors of the sellar and parasellar region from January 2006 to December 2018 in our neurosurgical department. Forty patients were operated twice, and 7 patients underwent 3 surgeries over the described time period. Fifty patients were excluded as their histopathological results revealed sellar pathologies other than adenoma (meningioma, craniopharyngioma, hypophysitis, Rathke’s cleft cyst); in 145 patients, preoperative data or follow-up visual acuity was missing, therefore excluded from analysis (Fig. 2). Complete data on preoperative, postoperative, and 3 months follow-up visual acuity and fields was available for 191 patients (4 patients with microadenoma, 187 patients with macroadenoma). A total of 69% of the patients suffered from preoperative impairment of visual acuity (*n* = 133), and 30.4% had intact visual acuity (*n* = 58).

Overall mean and median age were 55 years (range 20–86 years), and 59.2% (113/191) were male and 41.8% (78/191) female. Analyzing the subgroup of patients with impaired and intact visual acuity, mean age was 50 and 58 years, respectively (*p* = 0.001). Table 1 describes the demographic values of the study populations depending on the visual function. Overall median preoperative Karnofsky Index was 90% (interquartile range 80–90%).

**Table 1:** Patient population with mean and median age, sex, visual field defects, and Karnofsky Performance Status Scale (KPS), depending on visual acuity impairment, **p < 0.001

	No impairment (*n* = 58)	Visual acuity impairment (*n* = 133)	*P* value
Age (years)			0.001**
Mean + SD	50 + 16	57.5 + 14.5	
Median	49.5	57	
Range	20–84	23–86	
Sex (n)			0.523
Male	55.2% (32)	60.9% (81)	
Female	44.8% (26)	39.1% (52)	
Visual field deficit			
Yes	27.6% (16)	70.7% (94)	0.000**
With bitemporal hemianopsia	10.3% (6)	34.6% (46)	0.001**
KPS (range)			
Median	90% (80–100)	90% (40–100)	0.000**
Tumor volume	6.6	11.6	0.002*

Mean tumor volume was 10.41 cm^3^ (range 1.18–52.14 cm^3^, standard deviation of 9.51 cm^3^), with a median of 7.32 cm^3^ (interquartile range 4.36–13.71 cm^3^). The median Knosp grade was 3 (range 0–4), and most tumors were classified as Wilson-Hardy Class 2 (range 0–4). Inter-rater reliability showed an excellent agreement between the two raters (0.972, 95% confidence interval 0.757–0.991, *p* < 0.001).

### Preoperative visual field defects

In total, 110/191 patients had specific visual field defect such as complete or incomplete bitemporal hemianopsia (57.6%): perimetric findings of bitemporal hemianopsia were diagnosed in 52 patients (47.3% of all patients with visual field defects, 27.2% of all patients). A total of 54 patients had specific visual field defects without bitemporal hemianopsia (49.1% of all patients with preoperative field defects and 28.3% of all patients).

Unspecific visual field defect changes were registered in 33 patients (30% of all patients with visual field defects, 17.3% of all patients).

### Preoperative visual acuity

Mean preoperative visual acuity was 0.76 for the right and left eye (**R** and **L**).

Visual acuity impairment was found in 133/191 patients (69.6%). All patients suffered from pituitary macroadenoma. Mean visual acuity was 0.64 **R** and 0.65 **L**. Median Knosp classification score was grade 3, median Hardy classification grade 2. Preoperative visual acuity significantly depended on the Knosp classification (*r* = − 0.192, *p* = 0.029) but not on the grading provided by Hardy at al. (*p* = 0.395).

### Surgical performance

Regarding the intraoperative parameters, 67% (128/191) of the patients were operated microsurgically and 33% (63/191) underwent an endoscopic approach. In 27.2% of the cases, intraoperative CSF flow was noticed (52/191).

Mean surgery duration was 80 min (range 24 to 320 min). GTR was achieved in 50.8% (*n* = 97) of the patients, partial resection in 64.2% (defined as more than 90% tumor resection, 122/191 patients).

### Clinical visual function outcome

One week after surgery, mean visual acuity improved from 0.64 **R**/0.64 **L** to a value of 0.72 **R** and 0.75 **L** in patients with preoperative impairment of visual acuity (*p* < 0.005) postoperatively. At 3 months follow-up, visual acuity continued to recover with a visual acuity of 0.76 **R** and 0.80 **L** (Table 2).

**Table 2:** Preoperative, postoperative, and 3-month results comparing data on visual acuity and visual field defects in patients with preoperative visual impairment. The stars (**) mark highly significant improvements of visual function (*p* < 0.001) and visual field defects (paired *t* test and McNemar test)

	PreOP	PostOP	3 Mo FU	Improvement pre/post OP	Improvement pre OP/3 months
Visual acuity					
With visual impairment					
**R**	0.64	0.72	0.76	0.08**	0.12**
**L**	0.65	0.75	0.80	0.10**	0.15**
Field defects in patients with visual acuity impairment	57.6%	42.4%	29.8%	15.2%**	27.8%*
Bitemporal HA	34.6%	23.3%	18.1%	11.3%**	16.5%**

We observed a minor deterioration of visual acuity in patients with intact preoperative visual function in 12.1% (maximum decrease to visual acuity of 0.9 in 7/58 patients), and 84.5% remained stable (49/58). Two patients showed a postoperative decrease but had a complete recovery until follow-up examination.

Regarding patients with impaired visual function, postoperative visual acuity improved in 41.4% and 46.6% for the right and left eye after surgery (44.4% **R** and 39.8% **L** remained stable). At 3 months follow-up, 48.2% **R** and 54.4% **L** had improved and 36% **R** and 30.7% **L** remained stable. The postoperative value, but not the recovery of visual acuity, significantly depended on the Knosp classification in bivariate and multivariate analysis (*p* = 0.004 **R** and *p* = 0.03 **L** vs. *p* = 0.952). We classified the visual acuity impairment in 3 groups: visual acuity below or equal to 1/10 (*n* = 10), visual acuity ranging between 1/10 and 5/10 (*n* = 34), and visual acuity above 5/10 (total *n* = 147, *n* = 89 if excluding patients with intact visual acuity). In the first group, only 14.3% improved after 3 months; in the second group, 66.7% had an increase of their visual acuity; and in the third group, 44.2% improved in patient with visual impairment but visual function above 5/10. The statistical analyses show a significant difference between the groups (*p* = 0.000).

### Prognostic factors

The strongest predictor for improvement of visual acuity at 3 months follow-up was age (*r* = − 0.29, *p* = 0.001 **R** and *r* = − 0.202, *p* = 0.019 **L**); Knosp and Hardy showed an influence on the visual acuity value but not recovery if controlled for age in multivariate analysis. Surgery duration and surgical technique did not influence visual acuity improvement in linear regression analysis (*p* = 0.113 and *p* = 0.603).

## Discussion

Our data suggest that transnasal transsphenoidal pituitary surgery is a safe and effective procedure for pituitary adenomas with a low rate of permanent complications and a satisfying postoperative outcome by improving the visual acuity significantly after surgical tumor resection and furthermore visual function after 3 months.

Most patients experienced an improvement or stagnation of their visual acuity after surgery in case of preoperative visual impairment. Our results are comparable with current literature, rating the improvement from 73 [16] to 67.5% [26] (pooled review data). Field defects improved in over 75% directly after surgery and more than 80% after 3 months, compared with current evidence (range 62 [16] to 81% [26]).

Limitations do occur through the retrospective nature of the study. Patient data was acquired and reviewed based on information available from the endocrinological, ophthalmological, and neurosurgical department as well as recruited from local practice doctors following patients after surgery. As mild visual field defects often go unnoticed [5], especially in older citizens [30], patients may suffer from visual impairment for several months to years. The duration of symptoms, as a subjective patient reported factor, can therefore be underestimating the timing of initial visual deterioration and influence the potential of visual recovery. A second limitation is the possible occurrence of comorbidities such as glaucoma and diabetes-related retinopathy and cataract. These entities have an impact on visual acuity and field defects [1], are often unrecognized in the elderly, and present a significant confounder in older patients [13]. In our study, we found a significant negative correlation between age and recovery of preoperative visual acuity impairment, which is most likely caused by coexisting comorbidities. Nevertheless, elderly patients did profit from surgical adenoma resection.

Follow-up data was recorded with a minimum time of 3 months. Although patient data was obtained from external outpatient records, a possible selection bias exists as patients tend to continue follow-up appointments in case of complicated postoperative courses. From 386 patients, only 191 could be included for further analysis due to a lack of complete data. As pituitary adenoma patients need visual function testing and endocrinological follow-up, patients may prefer to continue follow-up examinations closer to their place of residence. This inclusion of only 50% of the eligible patients presents a strong limitation.

Quality of life was not assessed in our presented study. In order to estimate the burden of disease and surgical treatment effect, patient-reported outcomes and perceived health should be included in further studies [33] to reflect the true effect of treatment.

We observed a trend towards a further improvement of visual acuity on the postoperative timeline, suggesting an ongoing recovery. These positive findings support the theory of time-dependent convalescence after tumor extirpation, congruent with current literature describing significant improvements after 6 months but not after 3 months [10, 25]. We found a significant recovery after short-time follow-up of 3 months; the overall treatment effect may therefore be underestimated compared with long-term analysis after 1 year. Unfortunately, we did not explore the visual recovery on a longer interval, but published data suggests even better results the later the analysis.

The last limitation of our study is the issue of minimum clinically important difference of visual acuity and visual field defects. Even though our patients had a statistically significant improvement of visual acuity, it does not answer the question of self-perceived subjective improvement. As visual impairment often goes unnoticed, we do not know if patients really experience their numerical increase of visual acuity or which value of increase subjectively impacts the patient. Unfortunately, no studies have answered the question which minimal amount of visual acuity leads to a subjective improvement in patients with pituitary adenoma. The only study identified, analyzing the minimum clinical difference in visual acuity, was conducted in older cataract patients and found a value of 0.41 [4]. This value seems rather high, and as described before, visual function changes in older patients may go unnoticed.

Our retrospective study focused on clinical factors predicting visual function recovery. Recent publications reported on more objective measurements to assess the prognosis, such as parafoveal and peripapillary perfusion [20], retinal nerve fiber layer thickness [21], and electrophysiological testing [19]. These measurements, together with clinical parameters and symptoms should be accounted all together to prevent irreversible optic nerve damage.

## Conclusion

In this study, we provide detailed data on the postoperative visual outcome of patients suffering from sellar tumors treated via TSS for visual impairment. Visual function seems to recover on a long-term basis, and visual acuity and visual field defects significantly improved after 1 week and continued to improve after 3 months. If possible, transnasal adenoma resection should be performed in all patients with preoperative visual impairment.

## Data Availability

The datasets used and/or analyzed during the current study are available from the corresponding author on reasonable request.
